# Functional Microorganisms Drive the Formation of Black-Odorous Waters

**DOI:** 10.3390/microorganisms12030487

**Published:** 2024-02-28

**Authors:** Yuchen Wu, Wenjing Wang, Xiaozhu Liu, Yanqing Sheng

**Affiliations:** 1 CAS Key Laboratory of Coastal Environmental Processes and Ecological Remediation, Yantai Institute of Coastal Zone Research, Chinese Academy of Sciences, Yantai 264003, China; 2 State Environmental Protection Key Laboratory of Land and Sea Ecological Governance and Systematic Regulation, Shandong Academy for Environmental Planning, Jinan 250101, China; 3 University of Chinese Academy of Sciences, Beijing 100049, China

**Keywords:** anaerobic, black-odorous water, Desulfobacterota, nitrogen, oscillator

## Abstract

Black-odorous waters are water bodies that are noticeably abnormal in color or emit unpleasant odors. River water pollution and ecological degradation have gradually emerged with urbanization and rapid economic development, and BOW has become frequent. The black-odorous evolution of urban water bodies is a serious environmental problem in many areas, posing a serious threat to both human health and the ecological environment. Functional microorganisms are closely related to the formation of black-odorous phenomena in water bodies, but the understanding of the mechanisms by which functional microorganisms influence the formation of BOW is very limited. In this study, water samples from the Guangdang River in Yantai, Shandong Province, China, were collected as the bacterial solution in the study, and how environmental factors and functional microorganisms affect the formation of black smelly water was investigated by artificially simulating black smelly water. The results indicated that different environmental factors have different effects on the formation of BOW. Anaerobic conditions accelerated the formation of BOW, and species diversity and species abundance were lowest under this condition. Hydraulic disturbance and nitrate effectively mitigated the BOW phenomenon, in which species diversity and species abundance were higher; controlling either of these variables was effective in mitigating the BOW phenomenon. Desulfobacterota played a key role in the formation of BOW, and reducing the proportion of Desulfobacterota in the microbial community could effectively improve the water quality. Possible directions of electron transfer in the process were hypothesized. This study contributes to identifying the biological driving factors for black-odorous evolution, presents insight for preventing BOW formation, and provides a scientific basis for subsequent BOW management.

## 1. Introduction

In recent years, many lakes and rivers have exceeded the maximum carrying capacity of their aquatic environment due to constant pollutant discharge, resulting in surface water quality deterioration worldwide [1]. In some areas, aquatic ecosystems have been seriously damaged, resulting in the appearance of black water and odorous sediment [2]. Black-odorous waters (BOWs) lose the resource availability of normal water bodies, and also affect urban landscapes, destroy aquatic ecosystems, and cause damage to human health. Generally, BOW is defined as water that exhibits an unpleasant color and emits an odor. However, the causes of BOW evolution are generally considered as the result of the interaction between physical, chemical, and biological factors [3]. BOW is the phenomenon of blackening and odorization of water bodies mediated by microorganisms under the combined influence of physical and chemical factors [4,5]. External inputs of excess organic matter, heavy metals, and nutrients, as well as disruptions in the biogeochemical cycles of nitrogen, phosphorus, and sulfur, can lead to the phenomenon of black odor in water bodies [6,7]. Organic pollutants will be decomposed under the action of aerobic microorganisms, consuming dissolved oxygen in the water, leading to a lack of oxygen in the water body, which, in turn, produces BOW [8].

Sulfate-reducing bacteria (SRB) play a key role in the evolution of water quality. Some volatile sulfur-containing compounds (such as methyl mercaptan, methyl sulfide, dimethyl disulfide, carbon disulfide, carbonyl sulfide, and hydrogen sulfide) are produced by SRB, which leads to irritating odors and is the main cause of malodor in water bodies [3,9,10]. SRB also produce S^2−^, which combines with Fe^2+^ and Mn^2+^ to form the metal sulfides FeS and MnS, which are the main constituents responsible for the blackening of water bodies.

Contemporary research on BOW mainly focuses on water quality monitoring and assessment [11], the mechanism of BOW formation [12], key thresholds [13], etc. Functional microorganisms are closely related to the formation of black-odorous phenomena in water bodies; however, little is known about the environmental conditions and functional microorganisms that drive the formation of BOW. The aim of this study is to investigate the growth of SRB under different conditions by analyzing the trends in physicochemical parameters and microbial communities, discussing the community structure and functional metabolic processes of microorganisms in the process of black-odorous formation, and gaining an in-depth understanding of the mechanism of formation of BOW, to provide a theoretical foundation and scientific basis for the management of BOW in the future.

## 2. Materials and Methods

### 2.1. Studying Site and Samples

Water samples were collected from Guangdang River (37°26′40″ N, 121°26′29″ E), located in Yantai, China. The river is polluted by factories and sanitary sewage along its bank [14]. Part of this river has become black and smelly, which heavily influences the lives of surrounding residents. Water samples were collected in polyethylene bottles and were taken back to the laboratory within 2 h. The samples were filtered through medium-speed filter paper and allowed to stand for 2 h. The supernatant was the mixed bacterial liquid of BOW. Bacterial solutions (BSs) were kept in sterile conical flasks and refrigerated at 4 °C for later determination.

### 2.2. Experimental Design

Artificially simulated BOW was used for the experiments, and the range of ionic species and agnates of the simulated wastewater was inferred from the method of Feng et al. [15,16]. The simulated BOW comprised 402 mg/L starch, 107 mg/L carbamide, 90 mg/L CaCl_2_, 50 mg/L Na_2_HPO_4_, 103 mg/L MgSO_4_·12H_2_O, 100 mg/L FeSO_4_·7H_2_O, and 10 mg/L KH_2_PO_4_. The reagents were analytical grade or above, and deionized water (Milli-Q) was used to prepare the solutions. To observe the actions of SRB under different conditions, four experimental groups and two control groups were established in this study (Table 1). The first group was the natural standing group (NS), which contained the raw water samples. The second group was an anaerobic group (AN), sealed with liquid paraffin to create an anaerobic environment. The third group, named OS, was treated with an oscillator at 140 rpm. The fourth group involved 30 mg/L of added NaNO_3_, and was called NS-N. The microbes in the waters were removed from water samples by filtration with a 0.22 µm filter membrane, which was considered a control check (CK) for NS, AN, and OS groups. Each treatment (2.5 L) was incubated in 28 °C thermostatic chamber.

### 2.3. Physicochemical Analysis

Dissolved oxygen (DO), pH, and oxidation–reduction potential (ORP) were measured using a YSI instrument (YSI Professional Plus, YSI Incorporated, USA) on site. The contents of nitrate nitrogen (NO_3_^−^-N) and chromaticity were detected by the ultraviolet spectrophotometric method [17]. Nitrite nitrogen (NO_2_^−^-N) was determined by a colorimetric method, coupling diazotized sulfanilamide with N-(1-naphthyl)-ethylenediamine dihydrochloride to form a reddish-purple azo dye [18]. Ammonium nitrogen (NH_4_^+^-N) was detected by salicylic acid spectrophotometry [18]. The concentration of sulfide was determined by the colorimetry of N, N-dimethyl-p-phenylenediamine [19]. The inhibition rate of sulfide was calculated with reference to Equation (1). Three replicates were conducted for each measurement, and blank samples as well as calibration curves were included in these quantitative analyses.

(1) $$X=\frac{\rho_{0}-\rho }{\rho_{0}}\times 100\%$$

V_0_: Content of sulfide in the control group, mg/L;

V: Sulfide content in the experimental group, mg/L;

X: Sulfide inhibition rate.

### 2.4. DNA Extraction, qPCR, and 16S rRNA Gene Sequencing

Water samples in the control and experimental groups were collected for bacterial community detection. The sample was filtered through a 0.22 µm filter membrane (Millipore, St. Louis, MO, USA). Total genomic DNA from samples was extracted using the CTAB method. The 515F-806R primer was used to amplify the V4 region of the 16S rRNA gene [20]. All PCRs were carried out with 15 µL of Phusion^®^ High-Fidelity PCR Master Mix (New England Biolabs, Ipswich, MA, USA), 2 µM of forward and reverse primers, and approximately 10 ng of template DNA. Thermal cycling consisted of initial denaturation at 98 °C for 1 min, followed by 30 cycles of denaturation at 98 °C for 10 s, annealing at 50 °C for 30 s, and elongation at 72 °C for 30 s. Finally, the samples were heated at 72 °C for 5 min. Small fragment libraries were constructed according to the characteristics of the amplified regions. Sequencing libraries were generated using the NEB Next ⑥ Ultra™ Ⅱ FS DNA PCR-free Library Prep Kit (New England Biolabs, Ipswich, MA, USA) following the manufacturer’s recommendations, and indexes were added. The libraries were checked with Qubit and real-time PCR for quantification and a bioanalyzer for size distribution detection. Quantified libraries were pooled and sequenced on Illumina platforms according to the effective library concentration and amount of data required. After read splicing and filtering and operational taxonomic unit (OTU) noise reduction, the validated data were then subjected to species annotation and abundance analysis to reveal the species composition of the samples.

### 2.5. Statistical Analysis

Sequence analysis was performed using Uparse software (Uparse v7.0. 1001, [21]. Sequences with ≥97% similarity were assigned to the same OTUs. In order to analyze the diversity, richness, and uniformity of the communities, alpha diversity was calculated from 5 indices in QIIME1 (Version 1.9.1): observed OTUs, Chao1, Shannon, Simpson, and Good’s coverage. The functional predictions and functional classifications were based on the program functional annotation of prokaryotic taxa (FAPROTAX) [22]. The experimental data, rarefaction curve, relative abundance histograms, and species abundance clustered heatmaps were statistically analyzed, calculated, and plotted using Origin (Version 10.0.5, OriginLab Corporation, Northampton, MA, USA) and Excel.2019 software. The Mantel test (vegan package in R) delineates the relationship between physiochemical factors and microorganisms in water samples [23]. Through the R packages “dplyr”, “ggcor”, and “ggplot2”, correlation combination figures were generated to show the relationships between microorganisms in water samples and physiochemical factors.

## 3. Results

### 3.1. Physical and Chemical Indicators of Different Water Samples

**Figure 1:**
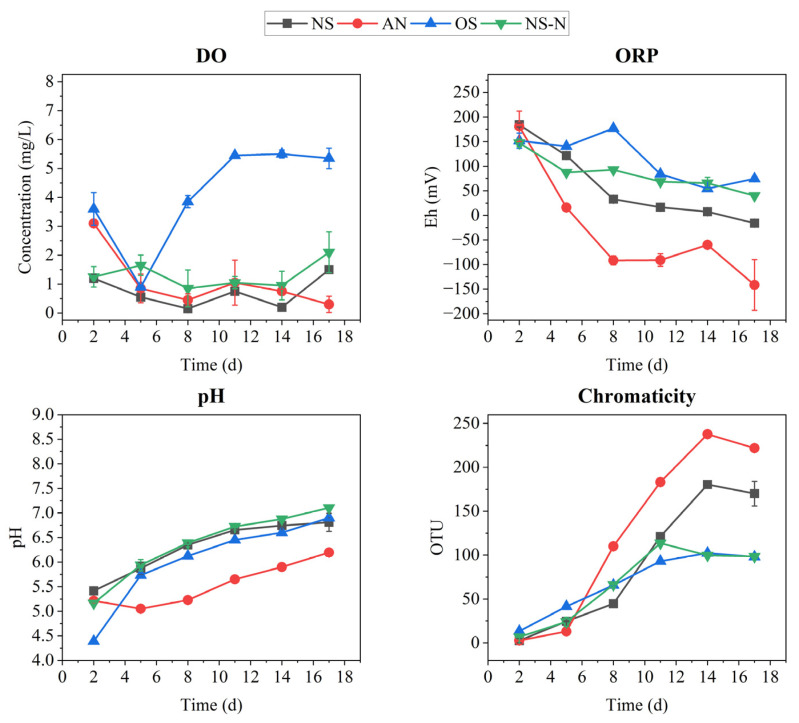
Change in DO, ORP, pH and chromaticity.

The working guidelines issued by China’s Ministry of Housing and Urban-Rural Development (MOHURD) in 2015 categorized BOW into two standards: mildly BOW and severely BOW. Mildly BOW has a transparency of 25.0–10.0 cm, DO of 0.20–2.00 mg/L, ORP of −200.00–50.00 mV, and NH_4_^+^-N content of 8.00–15.00 mg/L. Severely BOW bodies have a transparency of less than 10.0 cm, DO of less than 0.20 mg/L, ORP of less than −200.00 mV, and NH4^+^-N content of more than 15.0 mg/L. During the incubation, AN and NS already emitted odor at around day 5 and successively showed black materials around day 8 and day 11. The chromaticity of OS and NS-N was close to 100, and water samples were turbid, but there were no black substances or irritating odors in OS, and there was only a low concentration of black substances and irritating odors in NS-N. As shown in Figure 1, DO in NS reached a slightly black odor standard in 2 days, while in AN it remained below 2.00 mg/L for 5 days. The ORP in the AN and NS successively dropped below 50.00 mV on the fifth day, and ORPs for NS-N just reached the threshold for the mildly BOW criterion at the end. The DO of OS remained at a relatively stable state, except for a low value on day 5. The pH values in all groups dropped to 5.50 initially and gradually increased to 6.50 at the end of the observation. After 5 days of incubation, the coloration in NS and AN showed an abrupt increase, then maintained a state of heavy black odor (OUT > 100). However, there was no black color or odor in OS and NS-N, with a coloration value of approximately 90.

**Figure 2:**
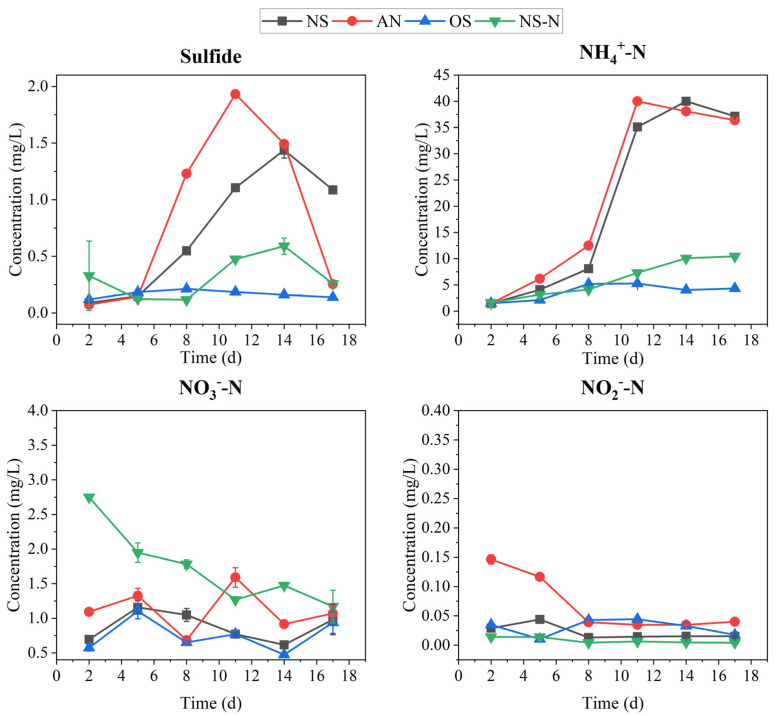
Changes in sulfide, NH_4_^+^-N, NO_3_^−^-N, and NO_2_-N in different tests.

The variations of sulfide under different incubation conditions are shown in Figure 2. The concentration of sulfide in NS and AN increased significantly after 5 days, and the black odor phenomenon was present with the increased sulfide. At the same time, NH_4_^+^-N levels in NS and AN reached the maximum (~40.00 mg/L), presenting a heavy black odor. The NO_3_^−^-N concentration in NS-N decreased constantly, while still presenting higher NO_3_^−^-N compared with other groups. Little NO_2_^−^-N was observed in AN at the beginning of the test, but then presented similar values to other treatments. After the fifth day, the sulfide content increased in the NS group, and sulfide inhibition rates in OS and NS-N were calculated according to Equation (1) using NS as a control. The inhibition rate of sulfide in NS-N varied from 56.97% to 78.77% from day 8 to day 17, while in OS it ranged from 59.50% to 88.50% from day 8 to day 17. 

### 3.2. Analysis of Bacterial Communities

Rarefaction analysis revealed that 64–388 OTUs were obtained from the samples. Alpha diversity, including Chao1, Shannon, and Simpson were calculated (Table 2). According to the Chao1 index, species abundance in each sample presented BS > OS > NS-N > NS > AN. Shannon indices in BS, NS, AN, OS, and NS-N were 5.112, 3.396, 1.985, 4.198, and 3.590, respectively, indicating that the highest species diversity was presented in OS, followed by NS-N and NS. The lowest species diversity was found in AN. In this experiment, changes in species diversity and species abundance exhibited consistency. The species diversity and species abundance of the AN group were severely lower than those of the other groups.

**Figure 3:**
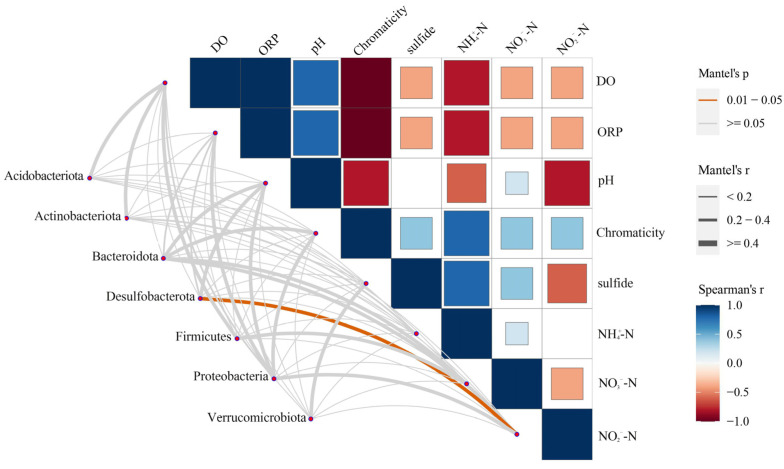
Heatmap of abundance clustering at the genus level and heatmap of predicted microbial function in water samples before and after incubation under different conditions. (**A**) Heatmap of microbial clustering at genus level under different culture conditions; (**B**) Heatmap of predicted microbial function under different culture conditions.

The phylogenetic relationships of microbial community were investigated, and representative sequences of the top 35 genera were obtained by multiple sequence comparison (Figure 3A). After incubation, the relative abundance of *Firmicutes* increased from an initial 0.53% to 3.30% to 16.27% under different culture conditions. The relative abundance of *Desulfobacterota* also increased. High percentages of *Ralstonia* (18.02%), *Delftia* (9.55%), and *Nevskia* (14.74%) were observed in BS. *Cupriavidus*, *Lacunisphaera*, and *Acinetobacter* were the dominant flora; the total percentage of these three increased from 0.48% to 32.10% in the total bacteria in NS, while in AN there were significant increases in *Azospira*, *Desulfovibrio*, and *Anaerospora*. In OS, *Allorhizobium*, *Taonella*, and *Acinetobacter* were dominant, with the total of the four accounting for 54.13%. In NS-N, *Burkholderia* (28.80%) and *Anaerospora* (16.17%) accounted for a larger proportion.

### 3.3. Functional Analysis and Mantel Test

Functional prediction was based on the functional annotations of the program based on the functional annotation of prokaryotic taxa (FAPROTAX) program. The clustered heatmap is presented in Figure 3B. From the identification results, microbial community functions demonstrated different succession under different environmental factors. In the initial water, the functional groups were mainly methanol oxidation, methylation, nitrite respiration, photoheterotrophy, phototrophy, and chloroplasts. The main functions of microorganisms in NS were transformed into aromatic compound degradation and predatory or exoparasitic. The main functions of the microorganisms shifted to nitrate respiration, nitrate reduction, nitrogen fixation, and nitrogen respiration, where the functional groups for sulfate respiration and respiration of sulfur compounds also appeared. Microorganisms in OS are extremely diverse, with hydrocarbon degradation, chitinolysis, and sulfate respiration functions. In NS-N, functional groups such as dark hydrogen oxidation and aerobic chemoheterotrophy were detected.

**Figure 4:**
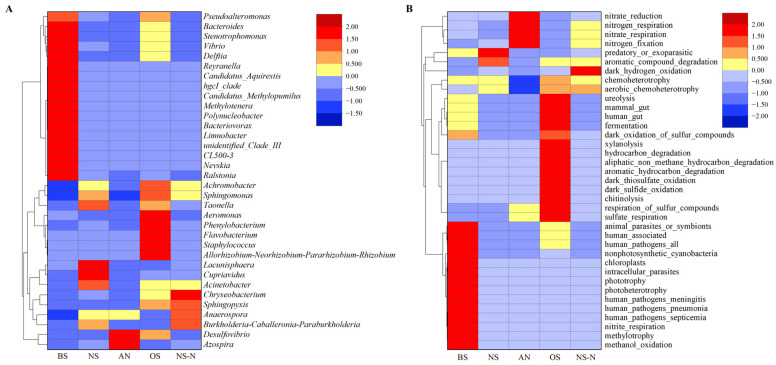
Percentage of metabolic pathways associated with the sulfur and nitrogen cycles.

**Figure 5:**
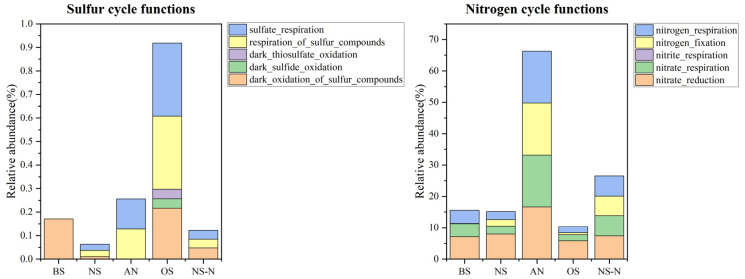
Relationship between bacterial community and environmental factors analyzed by the Mantel test. Spearman correlation indicated relation among environmental factors.

Function for the biogeochemical cycling of nitrogen and sulfur were screened using the KEGG database (Figure 4). Relative abundance of sulfur-related and nitrogen-related functional genera were all higher in AN than those in NS-N. Generally, microorganisms related to both sulfate and nitrate metabolism grew in anaerobic conditions. The relative abundance of sulfur-related functional genera in OS was higher than in other treatments, presenting more microbial diversity, and no black-odorous phenomenon was observed. In the presence of nitrate, the function of sulfate reduction decreased from 25.63% to 12.23%. Sulfate reduction was inhibited. The Mantel test was performed to discriminate predominant water quality parameters affecting microbes during blackening-odor formation (Figure 5). The results illustrated that NO_2_^−^-N had a significant correlation with Desulfobacterota.

## 4. Discussion

### 4.1. Anaerobic Condition Enhance BOW Formation

In the formation of BOW, the starch and carbamide were decomposed during the formation of a black odor in the water samples, in which the intermediate product of starch decomposition was pyruvic acid; the final products were carbon dioxide and water under aerobic conditions; and the final products were lactic acid or ethanol under anoxic conditions, resulting in low pH values [24]. In this process, aerobic microorganisms consumed DO in the water, resulting in anoxic water and the growth of anaerobic microorganisms [8]. The ORP of the water body decreased and took on a reduced state, where the microbials reduced sulfate and produced sulfide. This phenomenon was more pronounced in anaerobic environments. Acidic conditions promote the production of hydrogen sulfide, which gives the water an unpleasant odor. The presence of high concentrations of organic matter in the water creates conditions for the consumption of DO [25]. During the incubation process, with the growth of aerobic microorganisms, the DO in the water is gradually consumed, and anaerobic microorganisms such as SRB begin to grow. In the AN group, because the liquid paraffin isolated the water sample from the air, when DO in the water was consumed, the oxygen in the air could not enter the water, which enabled the water sample to form an anaerobic environment more quickly and promoted the growth and reproduction of SRB. NS was not completely insulated from the air; therefore, the black-odorous phenomenon appeared at a slightly later rate than that in AN. During the incubation process, SO_4_^2−^ was reduced to S^2−^, which reacted with Fe^2+^ to form FeS, causing the water sample to turn black, increasing the chromaticity of NS, AN, and NS-N. S^2−^ can volatilize as H_2_S to emit odor, and can also produce VOSCs through methylation to cause water samples to be odorous [9]. 

### 4.2. Disturbance and Nitrates Restrict BOW Formation

Compared with NS, OS showed better indicators of water quality. According to the standards published by MOHURD, NS reached severely BOW, but OS did not meet mildly BOW criteria. Interestingly, the OS retained a clear state throughout the experiment, and was found to have high DO concentration above 2 mg/L. This may be because the disturbance of water flow accelerates the mass transfer process of gases and promotes the dissolution of oxygen from the atmosphere into the water [26]. Under the disturbing effect, the contact area of the gas interface increases, and the mass transfer rate between oxygen and water increases, thus increasing the DO content. On the other hand, the rate of reoxygenation in the water column is greater than the rate of depletion, or is essentially equal, keeping the DO in the water at a high level [27]. NS-N reached a state of mildly BOW, which was less black than NS, suggesting that the addition of nitrate slowed the formation of BOW. However, it is not clear to what extent the nitrate content affects the mitigation of BOW; further experiments are still needed to explore this issue.

The level of DO was somewhat higher in the NS-N group compared with the NS group. The increase in DO level may be attributed to the fact that nitrate was used as an alternative electron acceptor to compete with oxygen for electron donors. It has been found that some autotrophic denitrifying bacteria of the phylum Ascomycota can use nitrate instead of oxygen as an electron acceptor to oxidize HS^−^, S^0^, and S_2_O_3_^2−^ [28]. The nitrate content in NS-N was decreasing over time, and less sulfide was produced in the NS-N group compared with NS, indicating that sulfide was oxidized while nitrate was consumed for denitrification, and the black odor phenomenon in the water body was slowed down. Proteobacteria was the dominant phylum in the NS-N group, accounting for 76.57% of the total number of bacteria. Haosagul found that some Proteobacteria could consume nitrate and oxidize sulfide to sulfate by denitrification, thus reducing the sulfide content in water, which is the same as the conclusion of this experiment [29].

### 4.3. Bacteria Enhance BOW Formation

In this study, a clear result was shown in that bacteria played a key role in BOW formation. In the control check group (CK), bacteria in samples were sterilized, and no BOW was observed. Rarefaction curves can reflect the depth of sequencing data; the curves tend to be flat, indicating that the amount of data is reasonable, and the sequencing data can accurately reflect microbial community structure of the samples. The sequencing species coverage was 100%, and the sequencing depth was sufficient to respond to the microbial change patterns of the samples. As shown in Table 2, the species abundance and species diversity of water in the non-BOW (OS) were significantly higher than those in the BOW groups (NS, AN, and NS-N). Firstly, the microbial community structure varied among groups. Proteobacteria was the dominant phylum of water microorganisms, regardless of whether the water body was polluted, which was consistent with previous findings [25]. The relative abundance of *Desulfovibrio* in AN increased during the blackening and odorization. *Desulfovibrio* belongs to the SRB group; a reducing environment is conducive to the growth of SRB [30,31]. Reduction in the abundance of *Desulfovibrio* and Desulfobacterota is key to inhibiting the occurrence of black odor in water bodies. On the other hand, species diversity, which increases the resistance of communities to environmental perturbations and enables the restoration of microbial functions after pollution, is key to ecological insurance [32]. Therefore, microbial diversity and community can improve the self-purification ability of water bodies, improve water quality, and effectively alleviate the deterioration of water quality in water bodies. The results of the Mantel test indicated that *Desulfobacterota* correlates with nitrite, and since *Desulfobacterota* are usually SRB, which are capable of reducing sulfate [33], it can be inferred that *Desulfobacterota* transfers electrons to nitrite in the process of reducing sulfate, and the electron transfer equation can be speculated as shown in Equation (2). As NO_2_^−^-N continued to participate in the denitrification reaction, it was found at lower levels in water.

(2) $${3SO}_{4}^{2-}+{4NH}_{4}^{+}\to {3H}_{2}S+4{NO}_{2}^{-}+2H^{+}+4H_{2}O$$

## 5. Conclusions

This study investigated the influence of microorganisms on the black-odorous phenomenon of water bodies under various environmental conditions, and further understood the changing rules of physicochemical parameters and microbial community structure during the formation of BOW. Different culture conditions affected the water quality and microbial community structure. It was found that anaerobic conditions would reduce species diversity and abundance and accelerate the formation of BOW, while hydraulic disturbance and nitrate incorporation effectively mitigated the black-odorous phenomenon in the water bodies. Controlling one of the two variables can effectively inhibit the BOW. The increase in species diversity and abundance can enhance the stability of the water body, improve the self-purification ability of water bodies, improve water quality, and effectively mitigate the deterioration of water quality. The Mantel test showed a correlation between Desulfobacterota and changes in NO_2_^−^-N, from which the direction of electron transfer in the presence of Desulfobacterota was deduced. Notably, the theoretical direction of electron transfer was hypothesized in this study, and further experiments are still needed to verify the hypothesis and to obtain more scientifically rigorous conclusions. For the nitrate addition treatment, more experiments are also needed to discuss the concentration range that is most suitable for the suppression of BOW. From a sustainable point of view, the use of microorganisms to improve water quality and restore water bodies is a long-term and effective means of governance, and the results of these studies can help elucidate the formation mechanism of BOW and provide a theoretical basis for the biological approach to the governance of BOW.

## Figures and Tables

**Table 1:** Various treatments on artificially simulated BOW.

Groups	CK	NS	AN	OS	NS-N
Water	2.5 L	2.5 L	2.5 L	2.5 L	2.5 L
Remove bacteria	+	-	-	-	-
Anaerobic treatment	-	-	+	-	-
Oscillator	-	-	-	+	-
NaNO_3_	-	-	-	-	+

**Table 2:** Alpha diversity in water samples before and after incubation.

Sample Name	Good Coverage	Observed Species	Chao1	Shannon	Simpson
BS	1.000	388	392.634	5.112	0.925
NS	1.000	85	85.000	3.396	0.858
AN	1.000	64	65.000	1.985	0.590
OS	1.000	387	387.955	4.198	0.883
NS-N	1.000	86	86.000	3.590	0.882

## Data Availability

All relevant data are available within the manuscript.
